# The Role of Plant-Associated Microbes in Mediating Host-Plant Selection by Insect Herbivores

**DOI:** 10.3390/plants9010006

**Published:** 2019-12-18

**Authors:** John M. Grunseich, Morgan N. Thompson, Natalie M. Aguirre, Anjel M. Helms

**Affiliations:** 1 Department of Entomology, Texas A&M University, College Station, TX 77840, USA; johngrunseich@tamu.edu (J.M.G.); mthompson@tamu.edu (M.N.T.); 2 Ecology and Evolutionary Biology Program, Texas A&M University; College Station, TX 77840, USA; n.aguirre@tamu.edu

**Keywords:** beneficial microorganisms, phytopathogens, herbivore foraging, oviposition, visual cues, olfactory cues, gustatory cues, vector herbivore, non-vector herbivore

## Abstract

There is increasing evidence that plant-associated microorganisms play important roles in shaping interactions between plants and insect herbivores. Studies of both pathogenic and beneficial plant microbes have documented wide-ranging effects on herbivore behavior and performance. Some studies, for example, have reported enhanced insect-repellent traits or reduced performance of herbivores on microbe-associated plants, while others have documented increased herbivore attraction or performance. Insect herbivores frequently rely on plant cues during foraging and oviposition, suggesting that plant-associated microbes affecting these cues can indirectly influence herbivore preference. We review and synthesize recent literature to provide new insights into the ways pathogenic and beneficial plant-associated microbes alter visual, olfactory, and gustatory cues of plants that affect host-plant selection by insect herbivores. We discuss the underlying mechanisms, ecological implications, and future directions for studies of plant-microbial symbionts that indirectly influence herbivore behavior by altering plant traits.

## 1. Introduction

Insects need food resources that provide sufficient nutrients for growth, development, and reproduction. Insect herbivores require food plants to fuel these processes and must forage to find suitable host plants within diverse ecological backgrounds [1,2,3]. To locate and assess the quality of potential host plants, insect herbivores typically rely on plant-produced cues that provide information about relevant plant traits [4]. An additional layer of complexity in herbivore foraging arises from plant and herbivore interactions with microorganisms. All plants associate with beneficial and pathogenic microbes and these microbes can play important roles in modifying plant traits that indirectly influence host-plant selection by insect herbivores [5,6]. For this review, we define insect herbivore forging behavior as the location and selection of food plants and we focus on studies evaluating host-plant preference or colonization. We also include measures of herbivore oviposition preference, as oviposition is a mechanism of host-plant selection by gravid females for future offspring [7,8]. Moreover, we also discuss the role of dispersal behavior and subsequent host-plant selection following herbivore contact with microbe-associated plants.

Insect herbivores are equipped with a range of sensory systems, allowing them to perceive and interpret information from their environment encoded as visual, olfactory, and gustatory cues (described in further detail below). Here, we focus on this subset of cues due to their prominence in the literature and importance in mediating host-plant selection by insect herbivores. Herbivores typically use plant-associated cues during foraging [4] and oviposition [9] as these cues can provide information related to plant location [10], identity, nutritional quality [11], and defensive status [7]. Cues from different sensory modalities often play different roles throughout the host-plant selection process, from initial location of plants or habitats [12,13] to selection of individual plants or tissues [10,14,15]. Many insect species rely on visual cues for locating plants over large distances, especially if they are capable of long-range dispersal [16]. In contrast, gustatory cues require plant contact and provide information about suitable tissues for feeding or oviposition [17]. The use of different cues varies among insect herbivore species [18] and particular cues may be more useful in certain habitats, like soil environments [19], or during certain times of the day, such as diurnal, nocturnal, or crepuscular activity [20]. Although visual, olfactory, and gustatory cues vary in relative importance during host-plant selection among different herbivore species and environmental conditions, these cues are often used in combination by foraging or ovipositing insects [15].

**Figure 1:**
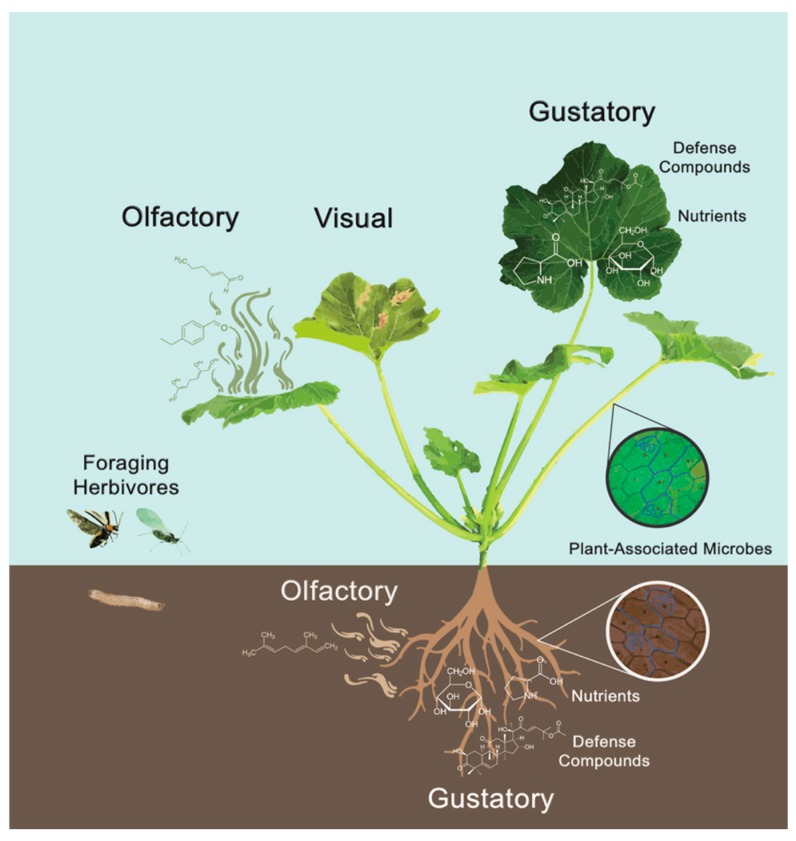
Beneficial and pathogenic microbes interact with above- and belowground plant tissues. These microbes can modify plant traits, such as visual, olfactory, and gustatory cues that insect herbivores use to locate and evaluate potential host plants. Plant olfactory cues are plant-produced volatile organic compounds. Plant visual cues are physical traits, such as plant size, shape, and color. Plant gustatory cues include nutrients, like sugars and amino acids, as well as plant defensive metabolites. Image by Alejandro J. Barroso, the figure is used with permission of the designer and has not been published elsewhere.

Interactions between plants and microbes are ubiquitous and can range from beneficial to parasitic or pathogenic. There is growing recognition that plant-associated microbes play important roles in modulating plant phenotypes and shaping interactions between plants and insects [21,22,23,24] For example, increasing evidence indicates that microbes alter plant-produced cues that subsequently influence the oviposition and foraging behavior of insect herbivores [25,26,27]. In this review, we discuss different ways that beneficial and pathogenic plant-associated microbes modify visual, olfactory, and gustatory cues in plants, focusing on microbes that spend at least a portion of their lifecycle on a plant. Furthermore, we examine how these microbe-mediated changes indirectly influence host-plant selection by insect herbivores (Figure 1).

## 2. Beneficial Plant-Associated Microbes

Plants often form mutualistic relationships with microorganisms. These beneficial plant-associated microbes interact with both above- and belowground plant organs and can live endophytically, within plant tissues, or ectophytically, depending on the species of microbe and the specificity or type of interaction [28]. Here we focus on beneficial soil bacteria, especially plant growth-promoting rhizobacteria (PGPR), including nitrogen-fixing *Rhizobia*, as well as beneficial fungi like arbuscular mycorrhizal fungi (AMF), and foliar and root endophytes, as these are among the best-characterized microbes mediating plant-insect interactions to date.

Beneficial microbes often alter plant growth or pest resistance traits that affect the performance and preference of insect herbivores. Microbes, like rhizobia or AMF, that increase plant nutrient acquisition, can also increase the nutritional quality of these plants for insect herbivores [29,30]. Moreover, the aptly named PGPR and fungi that enhance plant growth can provide greater amounts of available food resources for insect herbivores [31]. In contrast, certain species of beneficial microbes have also been observed to heighten plant defense responses via induced systemic resistance (ISR). ISR primes plants to mount faster or stronger defenses against a broad range of diseases or herbivores [32]. This differs from systemic acquired resistance (SAR), which is initiated by plant infection with pathogenic microbes (discussed below). For an extensive review of molecular mechanisms underlying ISR and how they contrast with SAR, we direct readers to [33]. ISR can enhance direct plant defenses, like toxic or repellent compounds, as well as indirect defenses, like volatile compounds or food rewards that attract natural enemies to kill herbivores. In this review, we limit our discussion of beneficial plant-associated microbes to their influence on herbivore foraging and oviposition. For a recent review of how beneficial plant-associated microbes alter insect predator and parasitoid behavior, see [34].

Through a range of mechanisms, beneficial microbes alter host-plant quality for insect herbivores, resulting in varied responses across different herbivore feeding guilds [35,36] or levels of specialization on particular host plants [37]. In addition to modifying insect performance on host plants, beneficial plant-associated microbes can also alter the foraging and oviposition behavior of insect herbivores [38]. Table 1 summarizes studies that include explicit tests of beneficial microbe-modified host-plant preference of foraging or ovipositing insect herbivores. Overall, few studies have directly tested beneficial microbe-mediated effects on insect herbivore preference, and even fewer have elucidated the cues responsible for these effects. Here, we review what is known about how beneficial microbes alter visual, olfactory, and gustatory cues in plants, highlighting ‘unknown’ cues as areas of future research, and we propose ideas to further our understanding of these tripartite interactions.

## 3. Pathogenic Plant-Associated Microbes

Plant-pathogenic microbes frequently cause disease symptoms that alter plant growth and/or chemistry and can influence the performance or behavior of insect herbivores. For example, plant pathogen infection often reduces plant growth [60,61,62] or causes color changes [63,64] or physical deformations to plant tissues [65]. Pathogen infection can also cause tissue damage that reduces photosynthate production which, coupled with the uptake of nutrients by the pathogen, can alter nutrient or resource availability for insect herbivores [66,67,68]. Plants respond to pathogen infection by activating physical and chemical defenses. This can include mechanisms to physically block or prevent the spread of infection, as well as production of antimicrobial compounds to fight the pathogen [69,70,71]. Plants typically tailor their defense responses to specific pathogens and activate different defense pathways or suites of defense traits against biotrophic (feeding on living plant tissue) or necrotrophic (feeding on dead plant tissue) phytopathogens. Plants exposed to biotrophic pathogens typically increase defenses through systemic acquired resistance (SAR), which is a physiological state of enhanced immunity against further infection in distal, uninfected plant tissues [72]. For an extensive review of molecular mechanisms underlying plant pathogen-mediated SAR, we direct readers to [73]. Plant-pathogen infection can reduce or enhance the performance of subsequent insect herbivores, depending on whether plant defense traits against the specific pathogen also confer resistance to insect herbivores, or suppress anti-herbivore defenses through crosstalk between defense pathways [74].

Pathogenic plant microbes have evolved to establish quickly and spread widely in plant populations. Some phytopathogen species are vectored by herbivorous arthropods, like insects, while others spread through abiotic factors like wind or water [26,75]. Vector-borne phytopathogens can be further characterized by their transmission types, depending on the time of feeding needed for the vector to acquire and transmit the pathogen (persistent, semipersistent, or nonpersistent), and whether the pathogen enters the hemocoel of its vector (circulative or noncirculative) [26,76,77]. A pathogen’s transmission strategy is often related to the nature of its interactions with herbivores. For example, some phytopathogen species, especially those that propagate within their vectors, can directly influence vector behavior or physiology [78,79]. For a recent review discussing the direct effects of pathogens on their vectors, see [26]. There is also accumulating evidence that insect-borne phytopathogens can have indirect, plant-mediated effects on insect herbivore behavior (Table 2).

In this review, we limit our discussion of phytopathogens to their plant-mediated effects on insect herbivore behavior. We focus on plant-pathogenic fungi, bacteria, phytoplasmas, and viruses, as these represent some of the best-characterized examples of phytopathogens influencing interactions between plants and insects. In Table 2, we summarize literature that measured the indirect effects of plant-pathogenic microbes on the foraging or oviposition behaviors of vector and non-vector insect herbivores. We review what is known about how plant pathogens modify visual, olfactory, and gustatory cues in plants, also calling attention to ‘unknown’ cues and outstanding questions in pathogen-plant-insect research to propel future investigation.

## 4. Visual Cues

Visual cues, in the form of patterns, dimensions, and spectral quality, are perceived by insect optical sensory systems [117]. Insect herbivores are equipped with compound eyes, ocelli and/or stemmata to detect visual cues, and use of these cues varies by species and eye morphology. Visual cues are light dependent and most commonly used by diurnal, aboveground organisms [118]. Insect herbivores use visual cues for both long- and short-range plant location [13] as well as for assessing plant quality [12]. Combinations of visual cues encoded as physical plant traits like size, shape, texture, reflectance, or color can convey a wide variety of information about plant location [119], nutrition [120], and defense status [121].

### 4.1. Influence of Beneficial Microbes on Plant-Produced Visual Cues

Visual cues for insect herbivores related to host-plant quality are predominantly influenced by beneficial plant-associated microbes through enhanced or reduced plant growth or biomass. In general, beneficial microbes are predicted to increase plant biomass through enhanced nutrient acquisition [31], decreased drought stress [122,123], or production of growth-related phytohormones [124,125]. Although the effects of beneficial microbes on plant growth and biomass are well-documented, surprisingly few studies have evaluated the influence of these effects on insect herbivore foraging and oviposition. Here, we highlight areas for possible future research by restricting our review to studies that considered the role of beneficial microbes in plant-insect interactions. For example, plant association with AMF was found to increase aboveground plant biomass by 87% across seven herbaceous plant species, and African cotton bollworm mass gain was higher on AMF-associated plants [126]. This suggests that foraging or ovipositing insect herbivores could benefit from detecting plants with AMF through visual cues like increased size to enhance their performance or fitness, although AMF-induced positive growth effects may be difficult to disentangle from other abiotic or biotic factors. Microbe-stimulated plant biomass gains are also not likely to affect host-plant discrimination by all species of foraging herbivores or in all contexts [43]. PGPR-stimulated plant biomass gains were correlated with reduced colonization of beetle herbivores in a field experiment with cucumber plants, although plant size was not likely the driving factor underlying these results [56]. Previous studies have also documented variation in plant responses to different species or isolates of beneficial microbes, which can affect insect foraging behavior. Recent work with strawberry demonstrated different AMF isolates had variable effects on multiple plant visual cues, including height, chlorophyll levels, and leaf thickness [44]. African cotton bollworm larvae preferred plants without AMF in detached leaf assays, however, in whole plant bioassays, they preferred the largest plants regardless of AMF status [44]. Overall, beneficial microbes can differentially alter plant growth and biomass, which can, in turn, influence the attraction or repellence of foraging insect herbivores in a context-dependent manner.

In addition to plant size, herbivores can also recognize physical plant defense structures, which affect host-plant selection. For instance, insect herbivores can recognize and clip plant trichomes to more easily access leaf tissues, although this behavior slows feeding and reduces insect performance [127]. Hence, herbivores may preferentially select plants producing fewer trichomes to increase foraging efficiency. Plant production of such physical defense structures as well as maintenance of microbial mutualisms can incur metabolic costs, indicating a potential trade-off for plants. A recent study found that tomato plants colonized by AMF had reduced trichome densities and increased herbivore performance [128]. Although not explicitly tested in this study, insect herbivores could potentially detect a decreased investment in physical defense structures in microbe-associated plants when making foraging or oviposition decisions to enhance their performance or fitness.

### 4.2. Influence of Pathogenic Microbes on Plant-Produced Visual Cues

Plant-pathogenic microbes often modify physical plant traits like size or shape that could provide visual cues for foraging or ovipositing insect herbivores. Plant pathogens also cause visible disease symptoms like mottled tissues [103,105,114], necrotic regions [85,114], and other color changes that serve as visual cues for insect herbivores [91,112,115]. Several vector-borne phytopathogens have been found to alter plant coloration in ways that enhance plant attraction to their insect vectors. For example, “flavescence dorée” phytoplasma causes yellowing in leaves of grape plants. In visual-based choice tests, leafhopper vectors preferred yellow, diseased plants over healthy, green individuals [97,98,129]. The spread of this pathogen depends on leafhoppers, and thus increased attraction to plant disease symptoms could increase pathogen transmission. In addition to phytoplasmas, several species of plant viruses (e.g., Luteoviridae) cause yellows diseases that result in yellowing of plant tissues [63,130]. Several studies have shown that aphids and whiteflies, which vector many species of viruses, are attracted to the yellow color caused by virus infection [64,116]. For example, aphids were attracted to visual symptoms of barley yellow dwarf virus on oat and barley in both field and laboratory experiments [115]. Another study reported that pea aphid vectors were attracted to yellowed leaves of fava bean plants infected with pea enation mosaic, bean yellow mosaic, or broad bean mottle viruses [131]. Aphids did not discriminate between healthy and infected plants when visual cues were removed, indicating that these viruses enhance vector attraction by modifying plant visual cues [103].

There is also evidence that non-vector-borne phytopathogens modify plant visual cues. For example, the fungal pathogen, *Phyllosticta paviae*, which induces visible necrotic regions on leaves of infected horse chestnut trees, influences the preference of a non-vector herbivore [85]. Ovipositing leafminers selectively deposited eggs on uninfected leaves and healthy portions of infected leaves, suggesting the necrotic tissue provided visual cues that reduced herbivore oviposition. Similarly, oviposition by light brown apple moths was lower on grape leaves infected by the necrotrophic fungal pathogen, *Botrytis cinerea*, and the rate of oviposition was inversely related to visual symptoms of infection [87]. Female moths may selectively avoid oviposition on infected plants to increase larval survival, as necrotrophic pathogens ultimately kill host-plant tissues. In contrast, another study reported that brown rice planthoppers preferred rice plants infected by the hemi-biotrophic bacterial pathogen, *Xanthomonas oryzae*. Attraction persisted at 15 days post-inoculation when visual disease symptoms were severe but olfactory cues of infected plants were not different from healthy plants, indicating visual cues played an important role in planthopper attraction [91]. As non-vector herbivores, foraging planthoppers may detect and capitalize on weakened defenses of infected plants for their own benefit.

## 5. Olfactory Cues

Olfactory cues are volatile chemical compounds that insects perceive using receptors located on olfactory organs, including the antennae, labial and maxillary palps, and ovipositor [132]. Most insect herbivores rely on olfactory cues from plants during at least one stage of the foraging process. Many insect species use plant-produced volatile compounds to locate and evaluate potential host plants [133] as these cues effectively transmit useful information over both short [134] and long [135] distances in a variety of environments. Olfactory cues can be general indicators of plant presence, for example the respiratory biproduct CO_2_ [19], or complex blends of volatile organic compounds (VOCs) that convey detailed information about plant identity [4], nutrient content [136], defense status, or risk of predation by natural enemies [137,138]. Plants emit characteristic blends of VOCs that vary by plant species, genotype, developmental stage, and tissue [139]. The production of plant volatiles is a dynamic process altered by pathogen infection, mechanical wounding or feeding by different herbivore species, resulting in quantitative or qualitative changes in volatile profile [139]. These induced VOC blends convey additional information to herbivores about changes in environmental conditions. Plant VOCs also play important roles in direct defense against herbivores and pathogens, as some volatile compounds have toxic or anti-microbial properties [140] or deter foraging or oviposition by herbivores [141]. Plant volatiles induced by herbivore or pathogen attack also provide indirect protection for plants by recruiting herbivore natural enemies [142] or beneficial microbes [143]. In summary, foraging or ovipositing herbivores interpret a diversity of information through olfactory cues to select acceptable host plants.

### 5.1. Influence of Beneficial Microbes on Plant-Produced Olfactory Cues

Plant associations with beneficial microbes can alter production of plant volatiles and modify host-plant selection by insect herbivores. For example, AMF associations with fava bean suppressed plant VOC emissions (specifically, napthalene, (S)-linalool, (E)-caryophyllene, and (R)-germacrene D) and increased attraction of aphids to plants with AMF [39,40]. Additionally, microbially altered plant VOCs can influence female herbivore oviposition. For instance, in tomato, root endophyte colonization quantitatively reduced VOC production—except for trans-β-caryophyllene, which plants produced in higher quantities when associating with endophytes—and resulted in increased cotton bollworm oviposition on endophyte-associated plants [45]. In contrast, PGPR association modified the VOC profile of maize plants, suppressing production of (E)-5-methyl-2-methylene-2-hexen-1-ol and decreasing European corn borer oviposition [54]. Foliar endophytes in perennial ryegrass also deterred host selection in female African black beetles, increasing 2-ethyl-1-hexanol acetate and decreasing dodecane emissions [51]. Another study reported no difference in constitutive VOC production by lima bean plants with *Rhizobia* compared to unassociated plants. However, following plant wounding, the VOC blend emitted by *Rhizobia*-associated plants differed from that of unassociated plants and was less attractive to Mexican bean beetles [59]. In addition to these explicit tests for foraging behavior and host-plant selection, we also highlight other studies which noted microbe-induced changes in plant VOCs and suggest these systems serve as avenues of future investigation on herbivore foraging and oviposition behavior [144,145,146,147].

In contrast to foraging by aboveground herbivores, soil-dwelling herbivores often rely primarily on olfactory cues to locate host plants [148]. Beneficial plant-associated microbes can alter belowground olfactory cues, which attract or repel belowground herbivores, depending on the interaction. For instance, an aboveground foliar endophyte of a grass hybrid increased belowground CO_2_ and suppressed root volatile emissions, repelling a foraging root herbivore [52]. PGPR associating with maize roots were recently shown to alter root VOC profiles, including E-β-caryophyllene production [149,150]. However, maize roots only enhanced production of E-β-caryophyllene following root herbivore damage, suggesting ISR-mediated priming of defenses in roots following herbivory [149]. The volatile compound, E-β-caryophyllene, is involved in host-plant selection by root-feeding western corn rootworm larvae, suggesting PGPR-colonized maize roots could be more attractive to subsequent herbivores [55,151]. Root herbivore reliance on olfactory cues indicates microbe-modified plant cues are likely to have a significant impact on belowground interactions.

### 5.2. Influence of Pathogenic Microbes on Plant-Produced Olfactory Cues

Olfactory cues from plants are frequently altered by pathogen infection, and these changes depend on the plant and pathogen species, as well as the progression of disease symptoms [152,153]. Plant production of volatile compounds may be modified by pathogenic microbes to influence vector behavior and benefit pathogen spread and can also affect the behavior of non-vector herbivores. For example, a non-vector species, European grapevine moth, avoided laying eggs on grape plants infected with the necrotrophic fungal pathogen, *Botrytis cinerea*, as infected plants emitted greater amounts of herbivore-repellent 3-methyl-1-butanol [86]. A similar experiment showed that beet armyworm moths, a non-vector of biotrophic rose powdery mildew, were repelled by volatiles from infected rose plants [80]. Another study reported that infection with anther smut fungus reduced floral VOCs (specifically, lilac aldehyde) in white campion flowers which deterred *Hadena bicruris* moths. These moths do not vector anther smut fungus. Their larvae, which are seed predators of white campion, have reduced performance when feeding on seeds of infected plants [83]. Based on the current literature, it appears that some species of non-vector herbivores detect pathogen-altered plant olfactory cues and avoid infected plants. This could benefit both the pathogen and non-vector herbivore through decreased competition for shared plant resources.

In contrast to phytopathogen interactions with non-vector herbivores, insect-vectored phytopathogens modify the olfactory cues of their host plants to increase vector attraction and enhance their transmission [26,27]. The first documented example of such manipulation revealed that potato plants infected with potato leafroll virus had altered VOCs that more strongly attracted the insect vector, green peach aphid, compared to uninfected plants [154]. Subsequent studies of other virus-plant-vector species combinations have reported similar findings of virus modification of plant VOCs with enhanced vector attraction to infected plants. This phenomenon has been observed for viruses with different transmission mechanisms including persistently, non-persistently, and semi-persistently transmitted viruses [34,99,102,108,109]. In addition to plant viruses, recent evidence suggests that insect-vectored bacterial pathogens also alter plant olfactory cues to enhance their transmission. For example, wild gourd plants infected with bacterial wilt emitted increased foliar VOCs (e.g., hexenal, E-2-hexenol, and ocimene) and reduced floral VOCs (e.g., 1,4-methoxybenzene). The insect vector, striped cucumber beetle, was more attracted to foliage of infected plants but dispersed to aggregate in healthy flowers, which increases bacterial transmission in this pathosystem [92]. Another study reported that citrus trees infected with the pathogenic bacteria, *Candidatus* Liberibacter asiaticus, produced a different blend of VOCs (specifically, increased methyl salicylate and decreased methyl anthranilate and D-limonene) than non-infected plants and were initially more attractive to the citrus psyllid vector [66]. This attraction was also observed in apple trees infected with the phytoplasma, *Candidatus* Phytoplasma mali. Infected apple trees released greater amounts of the compound E-β-caryophyllene which was highly attractive to the vector psyllid in field and laboratory experiments [95,96]. In general, these studies suggest that vector-borne phytopathogens commonly induce olfactory changes in plants that exaggerate existing host location cues to enhance vector attraction and increase subsequent pathogen transmission.

## 6. Gustatory Cues

Gustatory cues are non-volatile chemical compounds that insects perceive using gustatory receptors located on organs such as the antennae, mouthparts, tarsi, and ovipositor [155]. Insect herbivores often use plant gustatory cues to evaluate the nutrient content or defense status of potential host plants to make foraging or oviposition decisions [17]. Use of plant gustatory cues by herbivores in terrestrial environments requires physical contact and is typically involved in assessment of plant quality following initial location [156]. Plant gustatory cues are often altered by plant interactions with herbivores or microorganisms and thus provide herbivores with ecologically relevant information related to plant quality [157]. Examples of gustatory cues commonly used by insect herbivores include plant defensive secondary metabolites [158] or plant nutrients [159] like sugars and amino acids. Furthermore, we recognize that herbivores often detect gustatory cues through feeding, which itself damages plant tissues, introduces oral secretions, and triggers changes in plant metabolites [160]. Therefore, we predict that interactions between microbe-altered and herbivore-induced gustatory cues will frequently occur.

### 6.1. Influence of Beneficial Microbes on Plant-Produced Gustatory Cues

Beneficial microbes can directly increase nutrient acquisition in plants, thereby enhancing the quality of food resources available for insect herbivores. For example, AMF association increased phosphorus and nitrogen levels in rice, which enhanced attraction of ovipositing female rice water weevils [42]. In another study, however, AMF-inoculated *T. vulgare* plants also had increased phosphorus and nitrogen concentrations, but this increase had no effect on aphid preference [43]. Associations with beneficial microbes can also alter the production of plant defense compounds, suggesting the possibility of interactions between plant nutrients and defense compounds that can influence herbivore host-plant selection. For instance, plant inoculation with AMF differentially altered plant nutrients (levels of nitrogen and phosphorous), as well as defense compounds (foliar cardenolides and latex exudation), depending on the species of milkweed (*Asclepias* spp.) [161]. A milkweed specialist herbivore, the monarch butterfly (*Danaus plexippus*), prefers to oviposit on plants with low levels of cardenolides, suggesting that AMF colonization has the potential to modify monarch oviposition preferences [162].

Microbe-altered plant defenses also deter or attract insect herbivores depending on their ability to physiologically process particular compounds. For instance, PGPR-associated cucumber plants had decreased levels of cucurbitacin C, a bitter defense compound produced by cucurbits [37]. Cucurbitacins, although toxic to most generalist herbivores, are attractive and stimulate feeding in some coevolved herbivore species like spotted cucumber beetles. Previous research suggests PGPR-mediated reduction of cucurbitacin C, which reduced beetle feeding damage, could also decrease attraction in foraging or ovipositing beetles [56]. In contrast, another study reported that cotton plants (*Gossypium hirsutum*) treated with PGPR had increased levels of the defense compound gossypol and increased expression of genes that regulate its production, resulting in decreased performance of beet armyworm larvae on PGPR plants [163]. As a generalist herbivore, beet armyworm may avoid PGPR-associated cotton plants with increased gossypol that reduce its performance.

Recent evidence also indicates that beneficial microbes alter plant responses to herbivore damage, which may have cascading effects on insect herbivore behavior. For example, AMF-associated *P. lanceolata* plants differed in constitutive levels of chemical defenses depending on the AMF species. AMF-associated plants also had reduced induction of defense compounds (e.g., iridoid glycosides) following herbivory, which could influence host-plant selection by subsequent herbivores [164]. The continued exploration into species-level or genotypic variation in plant responses to beneficial microbes, and perhaps herbivores, will provide greater insight into the mechanisms driving host-plant selection by insect herbivores on microbe-associated plants.

### 6.2. Influence of Pathogenic Microbes on Plant-Produced Gustatory Cues

Pathogenic microbes modify plant gustatory cues through changes in defensive metabolites or plant nutritional quality. Altered levels of plant nutrients, including nitrogen, phosphorus, calcium, sugar, and amino acids, can influence host-plant quality for subsequent vector and non-vector insect herbivores [68,99,165,166]. For example, peanut plants infected with white mold fungus had elevated levels of soluble sugars and were more attractive to ovipositing beet armyworm moths [81,82]. Recognizing enhanced nutrient content in diseased plants suggests a general benefit for insect herbivores, including non-vectors, as plant-derived nutrients are essential for herbivore growth and development. However, studies of how plant pathogens affect gustatory cues used by non-vector herbivores are not well represented in the literature. We propose that gustation plays an important role in influencing non-vector foraging and oviposition on pathogen-infected plants and merits further study.

Similar to visual and olfactory cues, there are numerous examples suggesting vector-borne phytopathogens alter plant gustatory cues to modify vector behavior and promote their transmission success [66,99,101,167]. For example, infection of squash plants with cucumber mosaic virus (CMV) disrupted carbohydrate and amino acid ratios in phloem, and enhanced plant defense responses, reducing plant quality for the vector herbivore, green peach aphid [68]. Aphids detected these altered gustatory cues and rapidly dispersed to healthy plants after initial feeding on CMV-infected plants [99]. In another study, rice plants infected with tungro disease had increased free sugars and reduced soluble proteins. Vector leafhoppers preferentially fed on infected plants for up to 24 h before dispersing and settling on non-infected plants [101]. We note that gustatory cues primarily affected dispersal behavior in these systems, while initial host-plant attraction was typically mediated by changes in olfactory cues. Hence, pathogens may benefit from modifying suites of foraging cues that play different roles in vector attraction to infected plants and subsequent dispersal to healthy plants.

Plants co-infected with multiple vector-borne pathogens are a common occurrence in natural and agricultural ecosystems. In these cases, multiple pathogens may alter different cues within a single, shared host plant and change foraging behaviors of multiple vector species. One recent study investigated how soybean plants singly or co-infected with two plant viruses influenced plant attraction and palatability for two insect herbivore species. Soybean plants co-infected with bean pod mottle virus (BPMV) and soybean mosaic virus (SMV) were equally attractive to Mexican bean beetles and soybean aphids compared to healthy control plants. However, when plants were individually infected with either virus, the vector of BPMV (Mexican bean beetle) was more attracted to the virus-infected plants, which had higher levels of glucose. The vector of SMV, soybean aphid, was more attracted to SMV-infected, but not BPMV-infected plants, compared to healthy plants. This was correlated with lower levels of defense-related phytohormones (e.g., jasmonic acid) produced by SMV-infected and BPMV+SMV co-infected plants, altering plant attractiveness in a virus and vector-specific manner [110]. Although this is a single example, plant-pathogen co-infection is also likely to modify plant gustatory cues in other pathosystems and influence pathogen transmission dynamics.

## 7. Conclusions and Perspectives for Future Research

In nature, plants frequently interact with beneficial and pathogenic microorganisms. Here we reviewed the current literature and discussed different ways plant-associated microbes alter plant traits and indirectly influence plant interactions with insect herbivores. Both beneficial and pathogenic plant-associated microbes can modify visual, olfactory, and gustatory cues of their host plants in ways that affect the foraging and oviposition behavior of subsequent insect herbivores. Overall, our review revealed a limited number of studies have explicitly quantified the influence of plant-associated microbes on plant traits and the corresponding influence on herbivore host-plant selection. Among studies identifying specific plant cues mediating herbivore behavior, olfactory cues were most widely reported for both beneficial and plant-pathogenic species. This finding could reflect the relative importance of olfactory cues for mediating herbivore foraging decisions or could be the result of publication bias where many studies chose to focus on olfactory-based cues.

The majority of research in this area, to date, has focused on vector-borne phytopathogens altering plant cues for herbivore vectors. In general, vector-borne pathogenic microbes modified plant cues and the behavior of herbivore vectors in ways predictive of enhanced pathogen transmission, suggesting pathogen manipulation of both host plants and vectors (Table 2). On the other hand, non-vectored phytopathogens variably affected plant cues and insect herbivore behavior. Commonly, non-vector herbivore preference for infected or uninfected plants was correlated with herbivore performance on those plants. In contrast, beneficial plant microbes had inconsistent effects on plant visual, olfactory, and gustatory cues and the influence of these cues on herbivore behavior varied greatly among the combinations of microbe-plant-herbivore species studied (Table 1). Outcomes may vary so widely due to the facultative nature of plant interactions with beneficial microbes, dynamically oscillating to and from mutualism, which indirectly shape plant-insect interactions. We also note that very few studies have examined how plant microbes alter cues in belowground plant tissues and how these changes influence the behavior of soil-dwelling herbivores. Future research is needed to expand our current knowledge on the mechanisms of how plant-associated microbes indirectly influence herbivore behavior through modified plant cues, evaluating multiple plant cues to form a better understanding of these tripartite interactions.

Within the current literature, the majority of studies have focused on microbe-plant-herbivore interactions in agriculturally important crop plants and have rarely considered the influence of plant domestication or plant genetic variation on these interactions. Some notable exceptions include, a comparison of plant infection with potato leafroll virus in cultivated potato and wild solanaceous hairy nightshade plants. These studies found higher attraction of the vector herbivore, green peach aphid, to wild over cultivated plants, as well as increased attraction for virus-infected plants of both species [108,154]. Another recent study examined the effects of turnip yellows virus (genus *Tymovirus*) across a spectrum of domestication from cultivated false flax (*Camelina sativa*), a wild congener (*C. microcarpa*), and a hybrid of these two species. This study identified differences in plant susceptibility to virus infection and attraction of the vector, green peach aphid, among plant species [62]. In general, plant domestication is correlated with reduced plant resistance to herbivores, although there is not a clear pattern for differences in specific resistance traits among plant species [168]. This highlights the need for additional comparative studies of microbe-plant-herbivore interactions in domesticated plant species and their wild relatives to uncover broader patterns of how plant domestication affects microbially mediated changes in plant traits that influence herbivore behavior.

Most studies of microbe–plant–herbivore interactions to date have focused on tripartite interactions within controlled environmental conditions. A few exceptions include studies that have considered abiotic factors like soil nutrients [169] or drought stress [170]. There is abundant evidence that abiotic factors, such as water or nutrient availability [171], solar radiation [172], and temperature [173] influence plant physiology and defensive traits. Abiotically mediated changes in plant defenses affect the outcomes of plant interactions with beneficial and pathogenic microbes, in addition to herbivores. For example, if stressful abiotic conditions result in reduced plant defenses, plant-associated microbes might exert a stronger influence over plant phenotypes that affect subsequent herbivores. Alternatively, reduced plant defensive potential could result in reduced responsiveness of plant traits to microbial-induced changes, especially for olfactory cues like plant volatiles or gustatory cues like defensive metabolites. Moreover, abiotic conditions also disrupt plant interactions with beneficial microbes [23]. For example, plant-AMF associations shift from beneficial to parasitic in higher nutrient environments [174] and such shifts are likely to influence plant traits and subsequent interactions with herbivores. Future studies including abiotic variation are needed to better understand microbe-plant-herbivore tripartite interactions in a more realistic context and to gain insights into how such interactions might be affected in a changing climate [23].

Additional areas of microbe-plant-herbivore interactions that deserve more attention in future work are plant associations with multiple beneficial and/or pathogenic microbes, as well as the influences of insect-associated microbial symbionts. As discussed above, a recent study determined that co-infections or co-associations of multiple microbe species within a host plant are likely to affect the outcomes of herbivore foraging [110]. Additionally, although outside the scope of this review, insect herbivores often rely on microbial symbionts to overcome host-plant defenses [175], obtain nutrients [176], or biosynthesize nutrients the insect needs but the plant does not provide [177]. Future studies combining these distinct areas of microbial research (plant-associated and insect-associated) will further advance our understanding of the role microbes play in plant-insect interactions. We especially advocate for research on the interactive effects of plant-associated and insect-associated microbes on insect herbivore foraging and oviposition. Finally, future studies comprising a greater number and diversity of microbial and/or insect-herbivore species sharing a common host plant will provide a more realistic view of multipartite interactions and have the potential to reveal new ecological patterns within these interactions.

## Figures and Tables

**Table 1:** Beneficial Plant-Associated Microbes Modifying Plant Cues That Influence Insect Herbivore Foraging and Oviposition Behavior.

Beneficial Microbe	Plant Species	Insect Species	Cue	Effect on Insect Preference	Reference
**AMF**					
*Glomus* spp., *Rhizophagus irregularis, Gigaspora margarita*, *Paraglomus brasilianum*	Fava bean (*Vicia faba*)	Pea aphid (*Acyrthosiphon pisum*)	Olfactory, Gustatory	Attractive	[39,40]
*Rhizophagus irregularis*	Sweet pepper (*Capsicum annuum*)	Green peach aphid (*Myzus persicae*), western flower thrips (*Frankliniella occidentalis*)	Gustatory	Repellent, No Effect	[41]
*Glomus intraradices*	Rice (*Oryza sativa*)	Rice water weevil (*Lissorhoptrus oryzophilus*)	Visual, Gustatory	Attractive	[42]
*Glomus* spp.	Tansy (*Tanacetum vulgare*)	Green peach aphid	Visual, Gustatory	No Effect	[43]
*Rhizophagus irregularis* isolates	Strawberry (*Fragaria vesca*)	African cotton bollworm (*Spodoptera littoralis*)	Visual	Variable	[44]
**Root Endophyte**					
*Acremonium strictum*	Tomato (*Lycopersicon esculentum*)	Cotton bollworm (*Helicoverpa armigera*)	Olfactory	Attractive	[45]
**Foliar Endophyte**					
*Glomerella cingulate*	Tropical vine (*Merremia umbellata*)	Leaf beetle (*Chelymorpha alternans*)	Unknown	No Effect	[46]
*Neotyphodium coenophialum*	Tall fescue (*Lolium arundinaceum*)	Bird cherry-oat aphid (*Rhopalosiphum padi*)	Unknown	Repellent	[47,48]
*Neotyphodium* spp.	Alpine timothy hay (*Phleum alpinum*)	Bird cherry-oat aphid, Cereal leaf beetle (*Oulema melanopus*)	Unknown	Repellent, No Effect	[49]
*Epichloë* spp. *Neotyphodium* spp.	Multiple native grasses	Fall armyworm (*Spodoptera frugiperda*), American grasshopper (*Schistocerca americana*), Bird cherry-oat aphid	Unknown	Variable	[38]
*Acremonium loliae*	Perennial ryegrass (*Lolium perenne*)	Fall armyworm	Unknown	Repellent	[50]
*Neotyphodium lolii*	Perennial ryegrass	African black beetle (*Heteronychus arator*)	Olfactory	Repellent	[51]
*Neotyphodium uncinatum*	Grass hybrid (*Festuca pratensis* X *Lolium perenne*)	Root herbivore (*Costelytra zealandica*)	Olfactory	Repellent	[52]
*Neotyphodium* spp.	Numerous grass species	Black cutworm (*Agrotis ipsilon*)	Unknown	Repellent	[53]
**PGPR**					
*Bacillus* spp., *Fictibacillus* spp.	Maize (*Zea mays*)	European corn borer (*Ostrinia nubilalis*)	Olfactory	Repellent	[54]
*Bacillus* spp., *Fictibacillus* spp.	Maize	Western corn rootworm (*Diabrotica virgifera virgifera*)	Unknown	Variable	[55]
*Bacillus pumilus*	Cucumber (*Cucumis sativus*)	Striped cucumber beetle (*Acalymma vittatum*), Spotted cucumber beetle (*Diabrotica undecimpunctata*)	Visual	Repellent	[56]
*Paenibacillus* spp., *Bacillus* spp., *Brevibacillus* spp.	Bermudagrass (*Cynodon dactylon*)	Fall armyworm	Unknown	Repellent	[57]
**Rhizobia**					
*Bradyrhizobium* spp., *Rhizobium* spp.	Soybean (*Glycine max*)	Chewing and piercing-sucking herbivores	Unknown	Attractive	[58]
*Rhizobia* spp.	Lima bean (*Phaseolus lunatus*)	Mexican bean beetle (*Epilachna varivestis*)	Olfactory	No Effect	[59]

**Table 2:** Plant-Associated Pathogens Modifying Plant Cues That Influence Insect Herbivore Foraging Behavior and Oviposition Behavior.

Pathogenic Microbe	Plant Species	Insect Species	Vector Status	Cue	Effect on Insect Preference	Reference
**Fungi**						
*Podosphaera pannosa*	Rose (*Rosa chinensis*)	Beet armyworm (*Spodoptera exigua*)	Non-Vector	Olfactory	Repellent	[80]
*Sclerotium rolfsii*	Peanut (*Arachis hypogaea*)	Beet armyworm	Non-Vector	Olfactory, Gustatory	Attractive	[81,82]
*Microbotryum violaceum*	White campion (*Silene latifolia*)	Lychnis moth (*Hadena bicruris*)	Non-Vector	Olfactory	Repellent	[83]
*Melampsora allii-fragilis*	Willow (*Salix x cuspidata*)	Willow leaf beetle (*Plagiodera* *versicolora*)	Non-Vector	Unknown	Attractive	[84]
*Phyllosticta paviae*	Horse chestnut (*Aesculus hippocastanum*)	Horse chestnut leaf miner (*Cameraria ohridella*)	Non-Vector	Visual	No Effect	[85]
*Botrytis cinerea*	Grape (*Vitis vinifera*)	European grapevine moth (*Lobesia botrana*)	Vector	Olfactory	Repellent	[86]
*B. cinerea*	Grape	Light brown apple moth (*Epiphyas postvittana*)	Vector	Visual, Olfactory	Repellent	[87]
*Fusarium verticillioides*	Maize (*Zea mays*)	African sugar-cane borer (*Eladana saccharina*)	Vector	Visual, Olfactory	Attractive	[88]
*Puccinia punctiformis*	Creeping thistle (*Cirsium arvense*)	Weevil (*Apion onopordi*)	Vector	Unknown	Attractive	[89]
*Ophiostoma novo-ulmi*	American elm (*Ulmus americana*)	Elm bark beetle (*Hylurgopinus rufipes*)	Vector	Olfactory	Attractive	[90]
**Bacteria**						
*Xanthomonas oryzae*	Rice (*Oryza sativa*)	Brown rice planthopper (*Nilaparvata* *lugens*)	Non-Vector	Visual, Olfactory	Attractive	[91]
*Erwinia tracheiphila*	Wild gourd (*Cucurbita pepo* ssp. *texana)*	Striped cucumber beetle (*Acalymma vittatum)*	Vector	Olfactory	Attractive	[92]
*Candidatus* Liberibacter asiaticus	Citrus (*Citrus* spp.)	Asian citrus psyllid (*Diaphorina citri*)	Vector	Olfactory, Gustatory	Attractive then Repellent	[66]
*Candidatus* Liberibacter solanacearum	Potato (*Solanum tuberosum*)	Potato psyllid (*Bactericera cockerelli*)	Vector	Olfactory	Attractive then Repellent	[93]
*Xylella fastidiosa*	Citrus (*Citrus sinensis*)	Sharpshooters, leafhoppers (*Dilobopterus costalimai, Oncometopia facialis*)	Vector	Visual	Repellent	[94]
**Phytoplasmas**						
*Candidatus* Phytoplasma mali	Apple (*Malus domestica*)	Psyllid (*Cacopsylla picta*)	Vector	Olfactory	Attractive	[95,96]
*Candidatus* Phytoplasma vitis	Grape	Leafhopper (*Scaphoideus titanus*)	Vector	Visual	Attractive	[97,98]
**Viruses**						
*Cucomovirus* spp.	Squash (*Cucurbita pepo*)	Green peach aphid (*Myzus persicae)*, Melon aphid (*Aphis gossypii*)	Vector	Olfactory, Gustatory	Attractive then Repellent	[68,99]
*Cucumovirus* spp.	Squash	Squash bug (Anasa tristis)	Non-Vector	Unknown	Repellent	[100]
*Tunrgovirus* spp., *Waikavirus* spp.	Rice	Green rice leafhopper (*Nephotettix virescens*)	Vector	Gustatory	Attractive then Repellent	[101]
*Sadwavirus* spp., *Closterovirus* spp.	Red raspberry (*Rubus idaeus*)	Large raspberry aphid (*Amphorophora idaei*)	Vector	Olfactory, Gustatory	Attractive then No Effect	[102]
*Enamovirus* spp.	Fava bean *(Vicia faba)*	Pea aphid (*Acyrthosiphon pisum*)	Vector	Visual	Attractive	[103]
*Enamovirus* spp.	Pea (*Pisum sativum*)	Weevil (*Sitona lineatus*)	Non-Vector	Gustatory	Attractive	[104]
*Sobemovirus* spp., *Comovirus* spp.	Common bean (*Phaseolus vulgaris*)	Mexican bean beetle (*Epilachna varivestis*)	Vector	Unknown	Attractive	[105]
*Polerovirus* spp.	Potato	Green peach aphid	Vector	Olfactory	Attractive	[106,107]
*Polerovirus* spp.	Hairy nightshade (*Solanum sarrachoides*)	Green peach aphid	Vector	Olfactory	Attractive	[108]
*Luteovirus* spp.	Wheat (*Triticum aestivum*)	Bird cherry-oat aphid	Vector	Olfactory	Attractive	[109]
Comovirus spp., *Potyvirus* spp.	Soybean (*Glycine max*)	Mexican bean beetle, Soybean aphid (*Aphis glycines*)	Vector/ Non-Vector	Gustatory, Olfactory	Attractive	[110]
*Tobamovirus* spp	Tomato (*Solanum lycopersicum*)	Green peach aphid	Non-Vector	Unknown	Repellent	[111]
*Crinivirus* spp., *Begomovirus* spp.	Tomato	Silverleaf whitefly (*Bemisia tabaci*)	Vector	Visual, Olfactory	Attractive	[112]
*Potyvirus* spp.	Potato	Green peach aphid	Vector	Olfactory	Attractive	[113]
*Caulimoviruss* spp.	Turnip (*Brassica rapa*)	Turnip aphid (*Lipaphis erysimi*)	Vector	Olfactory	Attractive	[114]
*Luteovirus* spp.	Winter oat (*Avena* spp.), Winter barley (*Hordeum* spp.)	Rose-grain aphid (*Metopolophium dirhodum*), English grain aphid (*Sitobion avenae*)	Vector	Visual	Attractive	[115]
*Potyvirus* spp.	Soybean, Pepper (*Capsicum* spp.)	Green peach aphid, Corn aphid *(Rhopalosiphum maidis)*	Vector	Visual	No Effect	[116]
